# Polysaccharides and Phenolic Compounds Recovered from Red Bell Pepper, Tomato and Basil By-Products Using a Green Extraction by Extractor Timatic^®^

**DOI:** 10.3390/ijms242316653

**Published:** 2023-11-23

**Authors:** Mohamad Khatib, Lorenzo Cecchi, Maria Bellumori, Beatrice Zonfrillo, Nadia Mulinacci

**Affiliations:** 1 Department of NEUROFARBA, Pharmaceutical and Nutraceutical Section, University of Florence, Via Ugo Schiff 6, Sesto Fiorentino, 50019 Florence, Italy; mohamad.khatib@unifi.it (M.K.); maria.bellumori@unifi.it (M.B.); beatrice.zonfrillo@unifi.it (B.Z.); 2 National Interuniversity Consortium of Materials Science & Technology, Via Giusti 9, 50121 Florence, Italy; 3 Department of Agriculture, Food, Environment and Forestry (DAGRI), University of Florence, Piazzale Delle Cascine 16, 50144 Florence, Italy; lo.cecchi@unifi.it

**Keywords:** flavonoids, rosmarinic acid, pectin, DLS, ^1^H-NMR, acylation degree, methylation degree, *Ocimum basilicum* L., *Solanum lycopersicum* L., *Capsicum annuum* L.

## Abstract

Fruits and vegetables processing produces significant amounts of by-products rich in valuable bioactive compounds such as polyphenols and dietary fiber. Food by-product re-use promotes the eco-sustainability of several crops. This study aimed to apply green extractions of bioactive compounds from by-products of basil, tomato, and red bell pepper production. Tests were performed by applying extraction procedures both at laboratory scale and using the Timatic^®^ extractor. Water and ethanol 10% and 20% were used for extraction of red bell pepper and tomato, testing different temperatures (30, 50, and 90 °C; water at 90 °C and ethanol 20% were applied for basil. The obtained phenolic extracts were analyzed by HPLC-DAD-MS. Polysaccharides of tomato and red bell pepper were extracted at laboratory scale and chemically characterized using ^1^H-NMR to define the methylation and acylation degree, and DLS to estimate the hydrodynamic volume. Laboratory extraction tests allowed efficient scaling-up of the process on the Timatic^®^ extractor. Phenolic content in the dried extracts (DE) ranged 8.0–11.2 mg/g for tomato and red bell pepper and reached 240 mg/g for basil extracts. Polysaccharide yields (*w*/*w* on DM) reached 6.0 and 10.4% for dried tomato and red bell pepper, respectively. Dry extracts obtained using the Timatic^®^ extractor and water can be useful sources of bioactive phenols. The study provided new data on tomato and red bell pepper polysaccharides that may be useful for future applications.

## 1. Introduction

The processing operations of fruits and vegetables produce significant amounts of waste (approx. 25–60%) [1]. Within the European Union, approximately 700 million tons of agricultural waste are generated annually [2], nowadays known as food by-products [3,4,5]. Indeed, rather than waste, they are recognized as a cheap source of valuable bioactive molecules such as carotenoids, phenolic compounds, dietary fiber, and vitamins with several beneficial health attributes [6,7,8]. The recovery of these molecules might bring significant economic and environmental benefits and promotes a circular economy concept. Phytochemicals obtained from agri-food by-products can be used as dietary supplements, natural pigments, ingredients for formulation of functional foods, antimicrobials, and antioxidants in various industrial sectors (e.g., pharmaceutical, cosmetic) [6,8]. Food by-products promotion has become one of the most interesting trends for the eco-sustainability of several crops [7], with the final goal of production with almost zero waste. Their valorization involves at least some key steps, such as extraction optimization and chemical characterization of the extracts. 

Waste and by-products generated from tomato, red bell pepper, and basil are of high relevance as they arise in significant amounts in the European market. Waste of tomato, red bell pepper, and basil along supply chains includes products of an advanced or immature level of ripeness, defective or damaged fruits and leaves, and grading, sorting, storage, transportation, and processing waste, which can all be considered food by-products.

Tomato (*Solanum lycopersicum* L.) is the second most important vegetable crop next to potato worldwide, with annual production of 186 million tons fresh fruit produced in 2020 in 144 countries [9,10,11]. Tomato is rich in bioactive and valuable compounds such as carotenoids (mainly lycopene (80–90%) and β-carotene) in addition to vitamin C, vitamin E, and several phenolic compounds [10,11,12]. Tomato wastes were subjected to evaluation as a potential source of phenolic antioxidants and anticancer agents [10,12], identifying and quantifying some individual phenolic compounds, including phenolic acids, flavonoids, lycopene, and pigments [9,13]. 

Red bell pepper (*Capsicum annuum* L.) is one of the most important vegetables, widely cultivated worldwide and consumed as a fresh vegetable, condiment, pigment, and functional ingredient for human food, pharmaceutical, and cosmetic purposes [4]. Red bell peppers are rich in vitamin C and polyphenols, in particular flavonoids (e.g., quercetin and luteolin) and carotenoids [4,14,15] and is an excellent source of dietary fiber. Recently, polysaccharides from red bell peppers have showed potent anti-complement, immunomodulatory and chemo-preventive activities, and anti-inflammatory properties during in vitro and in vivo tests [16,17,18,19,20].

Basil (*Ocimum basilicum* L.) has been cultivated for over 1000 years in many regions of the world. It is widely used in folk medicine, and in cooking, perfume, food, and cosmetic industries. Some literature reviews shed light on the phytochemical components of basil, which have anti-inflammatory, chemo-preventive, anti-microbial, antioxidant, and cardiovascular properties, among others. According to several authors, rosmarinic acid (RA) is the main phenolic acid present in both flower and leaf tissues with lesser amounts of caffeic acid derivatives, such as chicoric acid [21,22,23,24]. RA is the most biologically active compound present in basil related to these activities [22,25]. Chicoric acid has been studied for its potential to inhibit HIV integrase, to enhance insulin secretion and glucose uptake, and to exert antioxidant activity [26,27,28,29]. Polysaccharides from basil are mainly present in seeds and have shown antibacterial, anti-inflammatory, and immunostimulant properties [30]. Moreover, basil seed mucilage has been used to formulate hydrogel films for wound dressing drug delivery [31]. 

The exploitation of these by-products through the recovery of bioactive compounds can help with achieving the sustainable development goals of the 2030 Development Agenda of the UN (https://sdgs.un.org/goals, accessed on 1 November 2023). As part of a circular economy, the use of green extraction techniques instead of conventional extraction methods is essential. Green extractions, as defined by [32], use bio-solvents (such as water, ethanol, or vegetable oils) and/or innovative technologies (e.g., ultrasounds, microwaves) in order to reduce the use of petroleum resources and energy consumption. The Timatic^®^ extractor follows these principles as it uses repeated percolation under controlled pressure, with water or hydroalcoholic solvent. The extraction alternates a dynamic phase, where a pressure is applied, to a static phase in which the extracted substances are transferred into the solvent. Timatic^®^ has been already applied in a patented process for the recovery of phenolic compounds from *Olea europaea* L. by-products [33]. The extractor has also been successfully used for the extraction of biocompounds from *Achillea millefolium* L. [34] and from the brown seaweed *Zonaria tournefortii* [35].

In light of the large amount of valuable by-products obtained during processing of fresh red bell pepper, tomato, and basil, it is considered increasingly important to recover bioactive compounds from them with the simplest and most environmentally friendly approach possible, with a view to their reuse for several applications [4,6,7].

The aim of this study was the optimization of a green extraction process for the recovery of bioactive compounds from by-products of the three agri-food matrices, namely red bell pepper, tomato, and basil. The objective was to define the best experimental conditions, evaluated in terms of phenolic extraction yields obtained with Timatic^®^, an extractor used by farms that produces the chosen by-products. The obtained extracts were dried, and their phenolic content was determined. At the same time, red bell pepper and tomato polysaccharides were extracted at laboratory scale and chemically characterized.

## 2. Results and Discussion

The objective was to collect experimental data useful for giving added value to fresh basil, tomato, and red bell pepper by-products provided by Fattoria Autonoma Tabacchi (FAT, Perugia, Italy). The selection of by-products was carried out by FAT, taking into account the amount of plant production by the company. The approach for tomatoes and red peppers was to extract the phenolic compounds from the dried material produced by a drying plant available in FAT. Preliminary results for basil indicated that a drying process at a temperature close to 50 °C (that applied for tomato and pepper) resulted in a significant loss of rosmarinic acid, probably due to phenoloxidase activity. In light of these observations, basil was extracted as fresh material.

A key step of this approach was to provide suitable laboratory-scale extraction methods, easily transferable to the Timatic^®^ extractor, reducing the use of organic solvents as much as possible. This system is characterized by repeated cycles of extraction and an applied pressure higher than the atmospheric pressure. Several laboratory-scale extraction tests were preliminarily performed to select the best combination of extraction temperature, time, and solvent, in order to reduce the number of experiments to be performed in the next step with the Timatic^®^ system. Samples have been characterized in terms of the main phenolic compounds by defining their content in the dry matrices and in the corresponding dry extracts. The results regarding phenolic profiles and contents are separately discussed below for each by-product.

Furthermore, polysaccharides obtained only on a laboratory scale from tomato and red bell pepper have been collected to define their structure and chemical composition, both of which have been little investigated so far. A better understanding of the structure of these polysaccharides can be helpful in defining the possible applications of these compounds.

### 2.1. Phenolic Compounds Characterization

To date, several extraction methods have been proposed in the literature to recover polyphenols from several agricultural by-products, and most of these processes use mixtures of water with a high percentage of organic solvents such as methanol, ethanol, or acetone [30]. In this study, we aimed to apply experimental parameters suitable to reduce both extraction costs and environmental impact, and to achieve a simple scaling-up of the extraction process. Therefore, to recover the phenolic fractions from the three matrices, the possibility of using 100% water or hydroalcoholic solutions with a low amount of ethanol (10% or 20%) at temperatures of 30 °C, 50 °C, and 90 °C was investigated. The same chromatographic method was applied to analyze the phenols from basil, tomato, and red bell pepper and the identification of the phenolic compounds was performed considering the literature data, comparison with pure external standards, retention times, and the UV–Vis and mass spectra.

#### 2.1.1. Phenolic Compounds in Basil

The identification of the main phenolic molecules in the samples was carried out in agreement with the literature [22,25], with the retention times and mass spectra of pure standards and using a lab-prepared standard of chicory leaves. Furthermore, the extract ion (EI) profiles for detecting the mass ions of the main phenols allowed extraction of the mass spectra of all cinnamic acids. All these data allowed confirmation of the presence of caffeic, chicoric, and rosmarinic acids as the main phenolic compounds in basil extracts with other minor cinnamic derivatives. An example of the chromatographic profiles of basil extract at 280 and 330 nm is shown in Figure S1. The quantification of their content was done using rosmarinic acid for expressing all the cinnamic derivatives (see Section 3.5).

Preliminary tests to control the concentration of rosmarinic acid indicated that during extractions with 20% ethanol, water at 30 °C, and water at 50 °C, the molecule was partially degraded. It was hypothesized that this degradation was determined by the polyphenol oxidases. To inhibit these enzymes, it was decided to choose the extract with ethanol 70% obtained from leaves previously blanched in boiling water for 30 s as a reference sample at laboratory scale. The enzymatic browning phenomena involving the phenolic compounds in basil leaves before and after the blanching can be observed in Figure S2.

**Figure 1:**
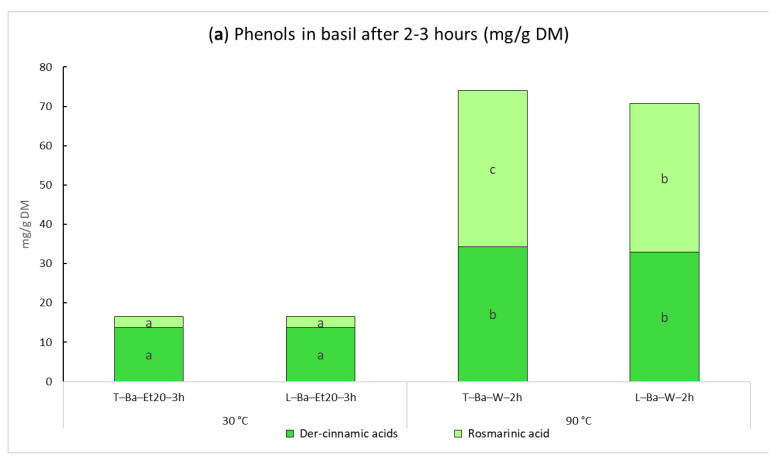

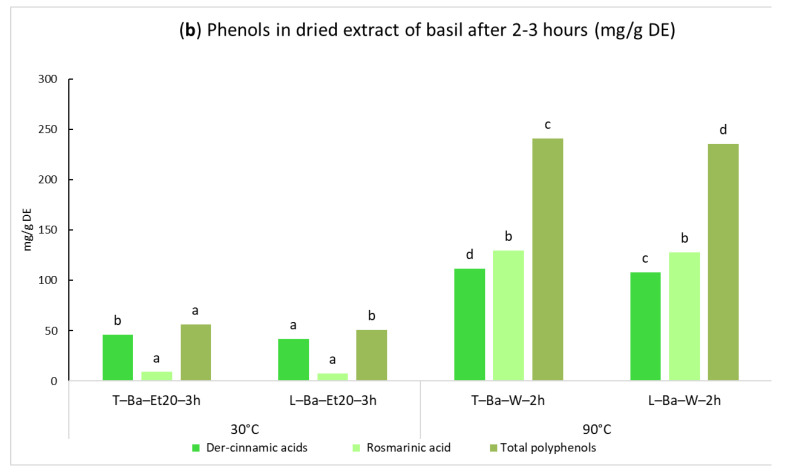
Extracts of fresh basil (Ba) obtained at laboratory scale (L) and by Timatic (T) with ethanol 20% (Et20), and water at 90 °C (W-90). The results are expressed in mg/g as mean of triplicates (**a**) on dried basil (DM) and (**b**) on dried extract (DE). For each series of data, different letters indicate significant differences at *p* < 0.05.

With Timatic^®^, 20% ethanol was used to evaluate whether the operating pressure (approximately 7 bar) and the reduced contact with oxygen during the extraction cycles in a closed chamber were able to counteract the activity of the phenoloxidases. The results regarding the Timatic extract (T-Ba-Et20-3h) and the corresponding laboratory-scale sample (L-Ba-Et20-3h) were very similar, confirming that the enzymes were not inactivated. The laboratory-scale screening carried out at 30 °C indicated that different extraction times did not affect the total phenolic content in the extract. On the other hand, working with hot water, the amount of phenols increased from 1 to 2 h of extraction (Figure 1). The best conditions to recover the phenols from basil using the Timatic^®^ extractor were obtained with water at 90 °C.

Regarding the quality of the dry extracts, the laboratory-scale reference sample (EtOH 70% from bleached leaves) reached 122.6 mg of total phenolic content with 73.2 mg of rosmarinic acid. A very interesting result is that the Timatic’s 90 °C water extract is much richer, with approximately double the concentration of these molecules (Figure 1b). The first tests carried out with EtOH 20% at 30 °C after three hours of extraction time gave a total phenolic content 4–5 times lower than the extraction with water at 90 °C carried out for only two hours (Figure 1b).

#### 2.1.2. Phenolic Compounds in Tomato

According to the literature [9,36], the main phenolic molecules detected in the tomato extracts were homovanillic acid, some gallic acid derivatives detected at 280 nm, and several flavonoids such as rutin-*O*-pentoside, rutin, and naringenin-*O*-hexoside (Figure S3). 

**Figure 2:**
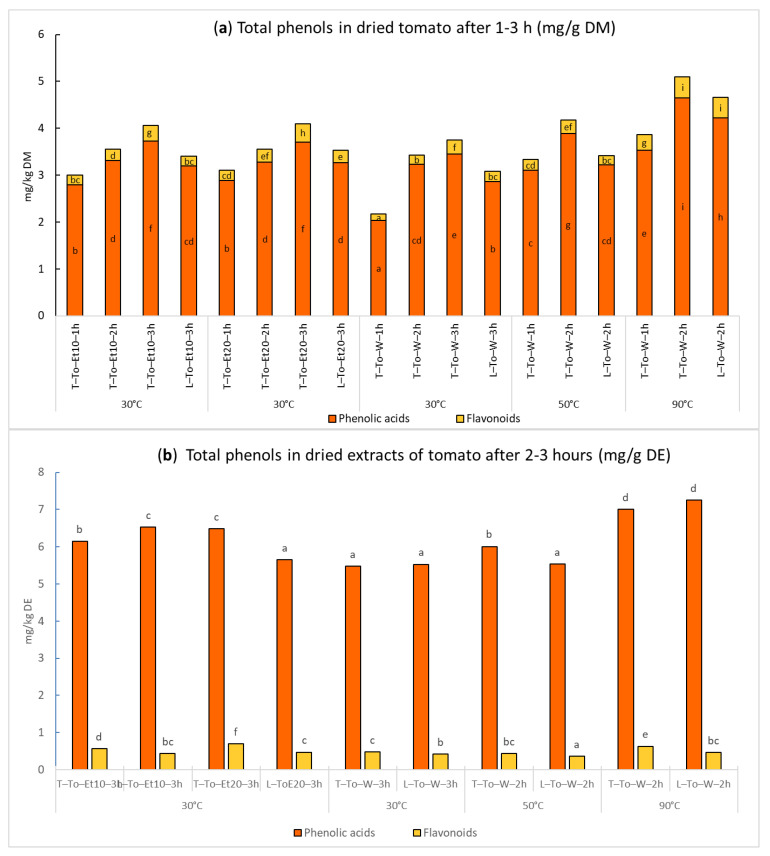
Phenolic content in several tomato extracts obtained at laboratory scale (L) and after extraction with Timatic^®^ (T) using different extraction times and temperatures. (**a**) Data obtained for dried matter; (**b**) data of dry tomato extract (DE) obtained at laboratory scale and by the Timatic extractor, expressed as mg/kg DE from triplicates. For each series of data, different letters indicate significant differences at *p* < 0.05.

The data in Figure 2a, expressed as mg on g of dried tomato matter, show that in all cases the extractions with Timatic^®^ lead to an increase in the recovery of phenolics, although not always with significant differences compared to laboratory-scale extraction. It is worth noting that Timatic^®^ samples obtained using only 10% ethanol gave the same results as the extracts produced using twice the amount of organic solvent. In particular, total phenols increased proportionally to higher temperatures and longer time (3 h). At the same time, the use of water at 30 °C gave lower yields, while water at 90 °C guaranteed the highest recovery of phenolic compounds, with no significant difference between laboratory-scale and Timatic^®^ samples. A longer extraction time of 3 h did not lead to increased phenolic concentration, and the maximum amount was reached for the T-To-W-2h sample (Figure 2a). Overall, these results indicated that it is possible to effectively simulate the extraction process of the Timatic^®^ system at laboratory scale for tomato, because using the same extraction mixture and the same extraction time, the results obtained with the two approaches were very similar.

The total phenolic content in the dried extracts ranged from 5.48 mg/g DE to 7.56 mg/g DE, confirming that the highest concentration was for the samples from water at 90 °C (Figure 2b), with the flavonoids as a minor class with respect to the phenolic acid derivatives.

#### 2.1.3. Phenolic Compounds in Red Bell Peppers

Several flavonoids were detected in red bell pepper, and their content ranged from 15% to 20% of the total phenolic amount. The identification of these flavonoids by means of DAD and MS detectors made it possible to identify the presence of several *C*-glycosides of luteolin and apigenin together with chrysoeriol 6-*C*-hexoside-8-*C*-pentoside and quercetin 3-*O*-rhamnoside-7-*O*-glucoside [20,37]. An example of the HPLC and EI profiles to detect specific flavonoids in the red bell pepper extracts is shown in Figure S4.

**Figure 3:**
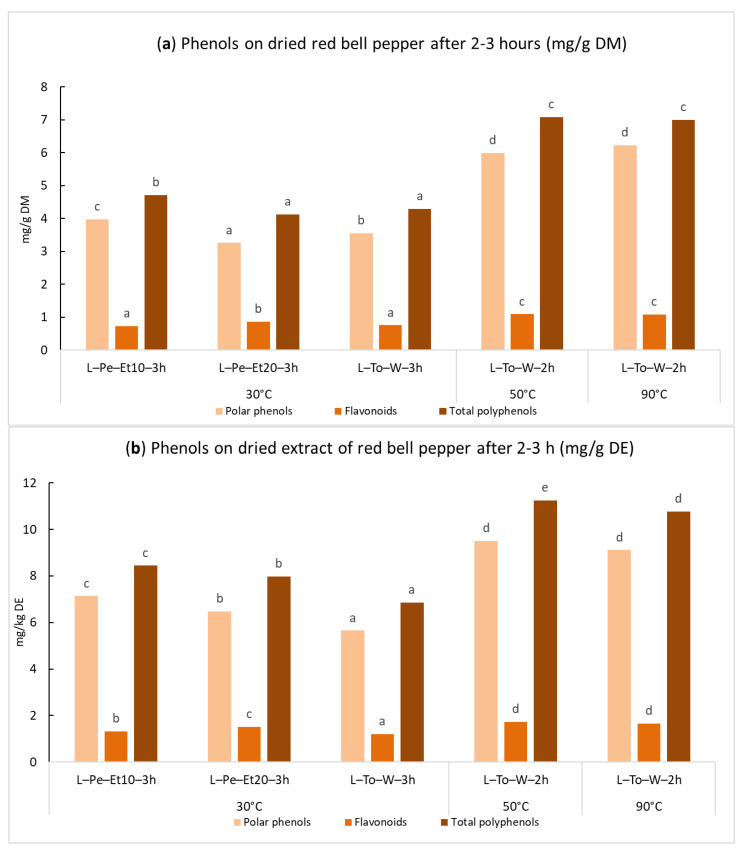
Phenolic content in the red bell pepper extracts expressed in mg/kg as mean of triplicates (**a**) on dried matter (DM) and (**b**) on dried extracts (DE). For each series of data, different letters indicate significant differences at *p* < 0.05.

In light of the previous results obtained for basil and tomato, which confirmed the possibility of simulating the extraction process of phenolic compounds at laboratory scale using Timatic^®^, only a small number of tests with this latter extractor were carried out. In particular, only the laboratory extracts with the highest content of phenolic compounds were reproduced with the Timatic^®^ system. Figure 3a shows that the total phenolic content was consistently lower with respect to basil extracts, but higher if compared to tomato extracts, ranging from approx. 4–7 mg/g DM.

As for the dry extracts, the maximum amount of total phenols at laboratory scale was 11.2 mg/g DE, reached with water at 50 °C (sample L-To-W-3h). The same extraction carried out with Timatic^®^ and water at 50 °C furnished 6.8 ± 0.2 mg/g on dried red bell pepper and 10.7 ± 0.2 mg/g on dried extract. Again, as already observed for basil and tomato, these values were close to the corresponding extracts produced at laboratory scale (Figure 3b).

### 2.2. Characterization of the Polysaccharides

The basil leaves were not used to extract the polysaccharides, because, according to the literature [30], they contain very little of these polymers (approx. 0.2–0.3% in dried leaves). Red bell pepper and tomato polysaccharides were evaluated because little data is presently available in the literature on their chemical characterization and structure. 

Some in vitro and in vivo studies described immunomodulatory and anti-inflammatwory properties for the polysaccharides from red bell pepper [16,17,18,19]. At the same time, few works have been focused on the polysaccharides extracted from the tomato fruit. The same authors, after a complex fractionation process and several steps of purification of the polysaccharides of tomato fruits, showed an in vitro hypolipidemic activity of some of the isolated fractions [38]. 

The molecular weight distribution of the polysaccharide was determined using dynamic light scattering (DLS), while ^1^H-NMR was used to determine the percentage of galacturonic acid and to measure the degree of methylation and acylation.

#### 2.2.1. Yield of Crude Polysaccharides from Red Bell Pepper and Tomato

**Figure 4:**
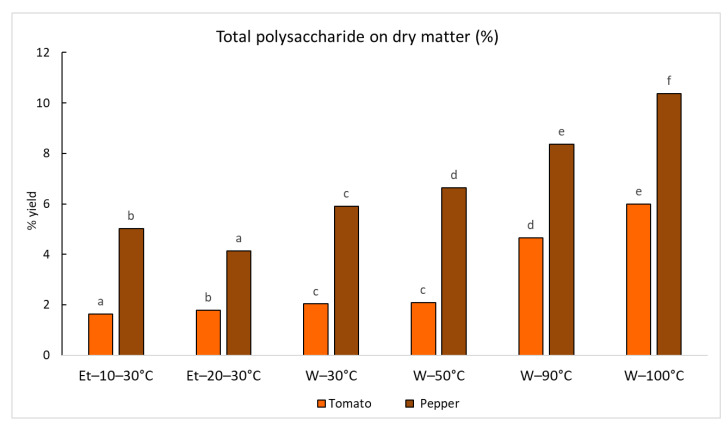
Percentage yields of total polysaccharides recovered after extraction with different solvents and successive precipitation with ethanol. The % yields are calculated without applying the dialysis. For each series of data, different letters indicate significant differences at *p* < 0.05.

After a preliminary screening carried out on the red bell pepper and tomato, the better conditions for the recovery of polysaccharides were defined according to the yields expressed on dry matter. The extraction carried out at 30 °C with ethanol at 10% and 20% was unsuitable for recovery of these molecules (Figure 4). The decoction was applied as a reference method to extract these molecules from both the two dry matrices, applying a drug/solvent ratio of 1/40 *w*/*v*. The polysaccharides yield showed the highest values in the samples obtained by hot water since the temperature is recognized as a determining factor for maximizing the extractability of these polymers, by increasing their water solubility [39]. A recovery of close to 10% on DM was reached for red bell pepper using water at 100 °C, while the maximum value for tomato was close to 6%. The crude polysaccharide fractions recovered from tomato and red bell pepper according to the experimental conditions reported in Figure 4 were then analyzed as described in the following.

#### 2.2.2. Characterization of the Polysaccharides by DLS and ^1^H-NMR

Dynamic light scattering (DLS) analysis was performed on polysaccharides from tomato and red bell pepper to obtain information on their average size and particle size distribution in water solution. Furthermore, the DLS algorithm was applied to estimate the mean molecular weight of the samples. DLS is commonly used for size characterization of various particles, proteins, polymers, carbohydrates, and gels [40,41]. Light dispersion was previously used to investigate the size of polysaccharides of palm fruit and potato and it allowed for the assessment of a wide range of molecular weights from 2.43 × 10^4^ g/mol to 1.87 × 10^6^ g/mol [42,43]. The data related to tomato and red bell pepper polysaccharides collected by the measurements with DLS are summarized in Table 1.

PdI is a mode of representation of the population size distribution of polysaccharides in water; it usually ranges from 0.0 for perfectly uniform particles/samples with a homogeneous size, to 1.0 for a highly diffuse samples with broad particle size clusters (https://www.malvernpanalytical.com, accessed on 1 November 2023). The PdI values of the polysaccharides of red bell pepper and tomato (Table 1) ranged from 0.21 (Pe-Et20-30°C) to 0.28 for the samples obtained by applying lower temperatures, indicating relatively narrow size distribution for these samples. The highest PdI values of 0.39 for tomato (To-W-90°C) and 0.35 for red bell pepper (Pe-W-90°C) resulted from extraction at the highest temperature. The use of water at 90 °C allowed not only recovery of a higher amount of polysaccharides, but also included those polymers that are less soluble at room temperature and have a higher molecular weight and different hydrodynamic volume in water.

For almost all the samples, the % area of the main peak (Pk1) of the polysaccharide sample was from 94% to 99%, with the only exception being for the samples extracted at 90 °C, which resulted in lower values (90–91%), in agreement with the results of PdI. 

The Z-PkI values are referred to the accumulated average size and are frequently chosen in DLS for assessing the hydrodynamic volume, providing an accurate measure of the mean size distribution of the major component/particle in solution. The Z-PkI values for both polysaccharide of red bell pepper and tomato showed similar sizes of 437–490 nm. The algorithm of the instrument allows estimation of the molecular weight associated to Z-Pk1, assumed to have polymers with prevalent linear structures and with few branched chains. The predicted molecular weight for PK1 ranged from 8.9 × 10^5^ KDalton (To-W-50°C sample), to 2.5 × 10^6^ KDalton (Pe-W-90°C sample). The estimated molecular weight of the minor component (Pk2) was lower and ranged from 1.3 × 10^4^ to 4.7 × 10^4^ KDalton.

#### 2.2.3. Galacturonic Acid, and Degree of Acylation and Methylation by ^1^H-NMR Experiments

**Figure 5:**
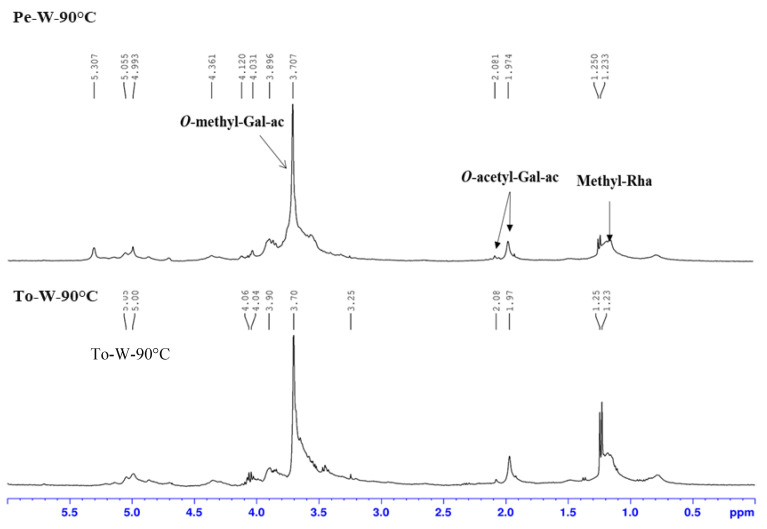
^1^H-NMR spectra in D_2_O of the polysaccharide samples. Pe-W-90°C (8 mg/mL) and To-W-90°C (7 mg/mL) after dialysis.

To date, little attention has been given in the literature to polysaccharides of tomato and red bell peppers, but the few available studies reported the presence of pectic polysaccharide both for tomato fruit [44] and pectin belonging to the group of rhamnogalacturonans from red bell pepper [17,18]. Taking into account the literature data, the presence of pectin was first evaluated by analyzing the proton spectra of the polysaccharide. The ^1^H-NMR spectra of two polysaccharide samples from red bell pepper and tomato are compared in Figure 5. The spectra are very similar, both with an intense singlet at 3.70 ppm that demonstrated the presence of several *O*-methyl groups linked to galacturonic acid units and typical of pectin structures. The presence of two singlets at 2.80 and 1.97 ppm indicated the presence of acetyl groups, while the methyl groups of the rhamnose units showed a doublet at 1.24 ppm. As for this latter signal, and according to previous results [44], different intensities were between the two spectra, indicating a higher percentage of rhamnose in the polysaccharides of tomato. The degree of methylation (DM) and acetylation (DA) of polysaccharides from tomato and red bell pepper were determined, as previously described [42,45], by working on dialyzed polysaccharides. The proton spectra of tomato polysaccharides before dialysis showed the presence of a lot of signals of monosaccharides at 3.00 ppm to 4.00 ppm.

**Figure 6:**
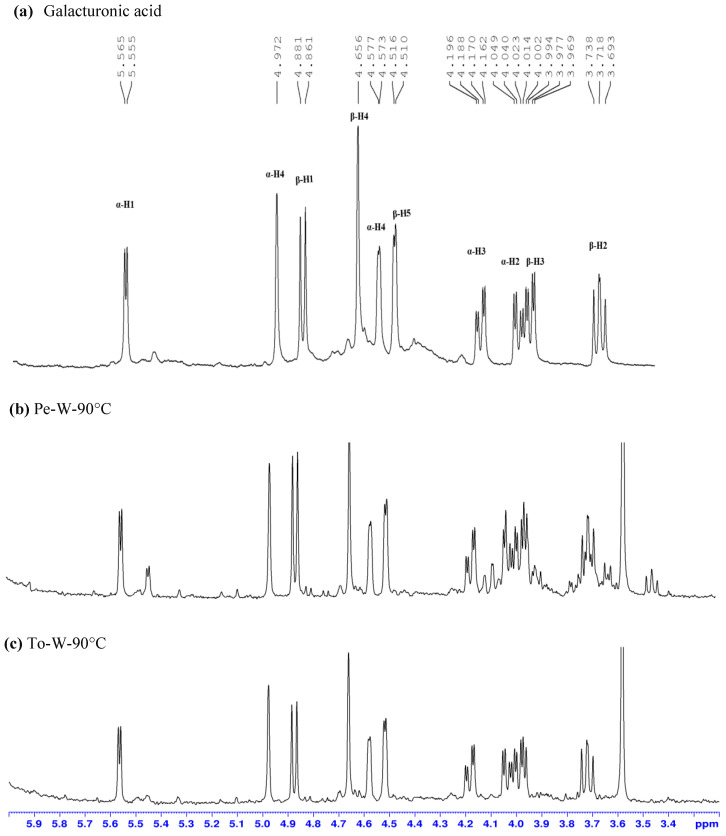
^1^H-NMR spectra of (**a**) galacturonic acid in D_2_O by 2M H_2_SO_4_; (**b**) hydrolysed Pe-W-90°C sample (5.3 mg/mL in 2 M H_2_SO_4_ at 100 °C); (**c**) hydrolysed To-W-90°C, sample (4.75 mg/mL in 2 M H_2_SO_4_ at 100 °C).

The acidic hydrolysis of the dialyzed polysaccharides (100 °C by 2M H_2_SO_4_) was conducted to determine the percentage of galacturonic acid and to confirm the presence of pectic structures. According to a method previously described [42,46], the determination was done by the integration of the anomeric protons α-H1 (5.55–5.56 ppm) and β-H1 (4.86–4.88 ppm) of galacturonic acid (Figure 6). The total amount of galacturonic acid in the polysaccharides of tomato ranged from 22.3% to 33.8% for the sample extracted in water at 90 °C. Higher percentages were found in the polysaccharides from red bell pepper with a maximum value close to 51% for both Pe-Et20-30°C and Pe-W-90°C samples.

The DM and DA were subsequently determined by ^1^H-qNMR after alkaline hydrolysis of the samples [42,47], measuring methanol and acetic acid using maleic acid as an internal standard; the data obtained for all samples are reported in Table 2.

DM and DA values in red bell pepper and tomato samples varied depending on the solvent and temperature. For tomato polysaccharides, the maximum DM was 71.00% for the To-W-90°C sample; much lower percentages were obtained for the other samples.

As for the polysaccharides from red bell pepper, the DM and DA values were higher and more homogeneous when compared with those of the tomato sample; the maximum value for DM was for the Pe-W-50°C sample, reaching 70.5%, and also with the highest DA value (21.57%). 

Both temperature and solvent significantly influenced DM and DA of the recovered polysaccharides, with a more relevant effect for tomato polysaccharides. Overall, the varying degrees of methylation and acylation in tomato and red pepper samples have implications for their functional and biological properties. Further research on these polysaccharides could help to better define their possible uses both in the food industry and for cosmetic formulations.

## 3. Materials and Methods

### 3.1. Materials

The dried by-products of tomato (*Solanum lycopersicum* L.), red bell pepper (*Capsicum annuum* L.), and fresh stems and leaves of basil (*Ocimum basilicum* L., lettuce leaves) were provided in September 2020 by an Italian farm: Fattoria Autonoma Tabacchi, from Città di Castello, (PG, Italy). The tomato (To) and red bell pepper (Pe) samples were approximately 5 kg each. The drying took place in FAT, in a ventilated oven: the products were placed on a conveyor belt with a max temperature of 50–55 °C. The tomato and red pepper samples were extracted without any preliminary cutting and the samples were irregular in size. The basil was a fresh sample containing stems and leaves coated with the extractive mixture within 24 h of collection.

### 3.2. Extraction of Phenolic Compounds

The following parameters were tested both at laboratory scale and with the Timatic^®^ (Technolab Perugia, Italy) extractor.

- Extraction time: 1 h, 2 h, and 3 h.
- Solvent: water, EtOH 10%, and EtOH 20%.
- Temperature: for the trials with hydroalcoholic solvents, the temperature was 30 °C, while for the trials with water, the temperatures were: 30 °C, 50 °C, and 90 °C.

The extractions at laboratory scale were carried our using 15 g of dried matter (drug/solvent ratio, 50 g/L) for the dried tomato and red bell pepper samples, while 10 g of sample were used for the fresh basil (drug/solvent ratio, 150 g/L). 

The extractions by Timatic^®^ (Technolab Perugia, Italy) were carried out with 440 g dried tomatoes or red bell pepper per 10 L of solvent, and 1.5 kg of fresh basil per 10 L of solvent. The extractions were performed using several extractive cycles (as required by the operation of the extractor) and each test was done by applying a matrix/solvent ratio of 1:20 (*w*/*v*) on dry matter basis. The samples were filled into the filter bag, which was closed and placed into the extraction chamber under controlled conditions of temperature, pressure, and time of solvent circulation. After a filling time of 60 s, and a pause of 30 s, the process started with a decompression of 6 min, a compression of 4 min, then several repeated extraction cycles (from 12 to 18) with a final time of 120 or 180 min. The applied pressures were from 7 to 9 bars and the emptying time was 2 min. The acronyms used for the extracts at laboratory scale and by the Timatic^®^ extractor are listed in Table 3.

### 3.3. Extraction of Polysaccharides

The extraction of total polysaccharides was only carried out at laboratory scale by working on dried samples of red bell pepper and tomato samples, both treated according to [39]. Briefly, a decoction of 1 h was carried out applying a ratio of 5 g/200 mL. The samples were then centrifuged at 5000 rpm for 10 min to remove the solid residue, then 2 volumes of ethanol were added to the recovered solution. The samples were stored for approx. 3 h at 0 °C to allow the precipitation of the polysaccharides, which were recovered after a new centrifugation (5000 rpm for 12 min at 0 °C). The precipitated polysaccharides were washed after being suspended in a minimum volume of 85% acetone, sonicated for 3 min using an ultrasonic cleaner at 40 kHz, collected after centrifugation at 0 °C, and lyophilized. The dried samples were analyzed by DLS and ^1^H-NMR.

### 3.4. HPLC-DAD-MS Analyses

The analyses of the phenolic extracts were performed using an HP 1200 liquid chromatographic system equipped with a degasser, a column oven, a binary pump, a DAD detector (Agilent Technologies, Palo Alto, CA, USA), and a Raptor ARC-18 column (150 × 3 mm, 5 μm, Restek S.r.l., Cernusco sul Naviglio, Milano (MI), Italy) with a pre-column of the same phase. The mobile phases were (A) 0.1% formic acid/water and (B) CH_3_CN. The following multistep linear solvent gradient was used: 0.1–5 min, 0–10—10% B (*v*/*v*); 10–20 min, 15–30% B; 20–25 min, 30–35% B; 25–28 min, 35–40% B; 28–31 min, 40–45% B; 31–37 min, 45–100% B; was then kept in these conditions for 5 min, and finally to 0% B in 5 min; total elution time was 47 min; flow rate 0.8 mL min^−1^ and oven temperature 28 °C. The UV–Vis spectra ranged from 200 to 500 nm and the chromatograms were acquired at 280 nm, 330 nm, 350 nm. The HPLC–MS analysis was done in both positive and negative mode using the same column and elution method previously described for the HPLC-DAD analyses; the HPLC was directly connected to an LTQ linear quadrupole ion trap mass spectrometer (Thermo Scientific, Bremen, Germany) via an ESI interface. The analyses were conducted in scan mode in the range 160–800 *m*/*z*, alternating between the two polarities. 

### 3.5. Quantification of Phenolic Acids and Flavonoids

The quantitative evaluation of phenolic compounds was performed using three external standards: gallic acid (purity ≥ 99%) at λ 280 nm (linearity range 0.0–2.4 μg, R^2^ = 0.9998) was used to quantify the more polar phenolic compounds in the three matrices. Rosmarinic acid (purity grade ≥ 99%) at λ 330 nm (linearity range 0.05–3.12 μg, R^2^ = 0.9998), was used to quantify rosmarinic acid and all the cinnamic acids in basil. The content of flavonoids was determined using a calibration curve of quercetin at λ 350 nm (purity ≥ 95%, linearity range 0–0.09 μg, R^2^ = 0.9995).

### 3.6. ^1^H-NMR Analysis

The ^1^H-NMR experiments were performed to study the polysaccharide samples using a 400 MHz instrument Advance 400 (Bruker, Bremen, Germany). The quantitative evaluation was done according to reference guidelines and applying the same protocol previously used for other matrices [42,48,49]. The polysaccharides from by-products of red bell pepper and tomato were dialyzed for 48 h at 5 °C in a nitrocellulose membrane with 12–14 k Dalton cut-off (Medicell Membranes Ltd., Greenwich, London, UK), and then freeze-dried. Each sample was of 4–6 mg of dry extract exactly measured and dissolved in 1 mL of D_2_O, with maleic acid added as internal standard (purity grade 98% from Merk Life Science S.r.l., Milano, Italy). 

The polysaccharides of tomato and red bell pepper after dialysis were used to determine the % DM and the % DA, according to the literature [42]. Approx. 4 mg of sample was exactly weighed in an NMR tube and 1 mL of NaOH 0.4 M in D_2_O was added, mixed and incubated at room temperature for 2 h. Successively, 20 µL of the internal standard maleic acid (5.1 mg/mL in D_2_O) was added to each sample before the ^1^H-NMR experiments. The % of acetic acid and methanol was evaluated through the integration of the *C*-methyl groups at 1.80 ppm and 3.22 ppm, respectively. The proton signal of the internal standard (6.24 ppm) was integrated as a reference signal for the purpose of quantifying according to the following formula:

$$C\;(\%)=\frac{I_{CH3}}{I_{mal}}\times \frac{N_{mal}}{N_{x}}\times \frac{{MW}_{x}}{{MW}_{mal}}\times \frac{W_{mal}}{W_{x}}\times P_{mal}$$

C (%), concentration of CH_3_COOH or CH_3_OH

I_mal,_ integral of the 2 protons of the maleic acid used as ISTD 

I_CH3_, integral of CH_3_ group of methanol or of acetic acid

N_x_, number of the protons of the methyl group of CH_3_COOH or CH_3_OH

N_mal_, number of protons for ISTD 

MW_x,_ mw of CH_3_OH (32 g/mol) or CH_3_COOH (60 g/mol)

MW_mal_, mw of the ISTD (116.1 g/mol)

W_x_, weight (mg) of dry polysaccharide 

W_mal_, weight of maleic acid

P_mal_, purity degree of the ISTD

Galacturonic acid was evaluated after acid hydrolysis by H_2_SO_4_ 2.0 M in D_2_O at 100 °C for 2.5 h. The content of galacturonic acid (Gal-ac) was calculated by the integration of the anomeric protons of the α-Gal-ac at 5.6 ppm and β-Gal-ac at 4.9 ppm and using the same internal standard. The formula previously cited was applied, considering as C % the concentration of Gal-ac; Nx, number of anomeric protons, one for α and β forms, respectively; MW_x,_ molecular weight of galacturonic acid, 194 g/mol.

The degree of methylation (DM) and acetylation (DA) were determined by the following formulas [47]:

DM (%) = (mol of methanol/moles of galacturonic acid) × 100

DA (%) = (mol of acetic acid/moles of galacturonic acid) × 100

### 3.7. Dynamic Light Scattering (DLS) 

The particle sizes of the polysaccharides were determined by DLS, Zetasizer Nano series ZS90 (Malvern Instruments, Malvern, UK), with a standard laser, 4 mW He-Ne, 632.8 nm, an optical fiber-based detector, and a digital LV/LSE-5003 correlator; the temperature controller (Julabo water bath) was set at 25 °C (kept constant by a Haake temperature controller). The time of correlation functions was analyzed by the ALV-60X0 software package (Malvern, version 7.2) to obtain the hydrodynamic diameter of the polysaccharides and the size distribution (polydispersity index, PdI). Autocorrelation functions were studied to determine the size distribution by fitting a single exponential to the correlation function. While the polydispersity values were determined for each peak as peak width/mean diameter. Scattering was analyzed in a cell of optical quality, with a capacity of 4 mL volume, by diluting the samples in concentrations of 1.25 mg/mL. For all the samples, the mean value of four measurements was taken at the constant level.

### 3.8. Statistical Analysis

All data were analyzed in-house using Microsoft Excel software (Microsoft 365 version) and presented as mean ± SD of at least a triplicate. The DLS data were obtained in triplicates and calculated by the Malvern, ver. 7.2 software package. The Excel software in combination with DSAASTAT software (v 1.1, Onofri, Pisa, Italy, 2007) was used to run one-way ANOVA and *F*-tests at *p* < 0.05, followed by LSD as the post hoc comparison used to identify significant differences among series of data.

## 4. Conclusions

The results described in this study highlighted the possibility of re-using some agricultural by-products by means of sustainable extraction processes to obtain extracts potentially suitable as new functional food ingredients. For all the investigated by-products, the extracts produced at laboratory scale showed similar features to those produced by the Timatic^®^ extractor. This finding is interesting because the study at laboratory scale can be proposed as adequately predictive of the results obtained with the higher capacity Timatic^®^ extractor already available on the market.

The study clarified that rosmarinic acid in basil leaves was stable at higher temperatures in water, but it was degraded at lower temperature even with a high % of ethanol in the extractive medium. This result suggested the presence of phenoloxidases which were also still active in the presence of organic solvents.

It is worth noting that water at high temperature resulted in a good solvent for maximizing both the phenolic concentration and the polysaccharide content in the extracts. This combination of bioactive molecules in the sample makes it possible to obtain a multi-functionalized extract, suitable, for example, for use as an antioxidant and gelling agent.

As for the polysaccharides from red bell pepper and tomato, further research will help to better define their possible uses both in the food industry and for cosmetic formulations.

## Figures and Tables

**Table 1:** Data from the DLS analyses of the polysaccharides from tomato (To) and red bell pepper (Pe), all obtained after 3 h of extraction. PdI, polydispersity index; D, diameter size; Z-Pk1, Z-average particle size of the main peak; Pk1 andPk2 the detected peaks in each sample; KDa, kilodalton; nd, for values below one unit; Est MW, Estimated molecular weight. For each series of data, different letters indicate significant differences at *p* < 0.05.

Sample	PdI	Z-Pk1	Pk1	Pk2	Pk1	Pk2	Est MW	Est MW
		D (nm)	D (nm)	D (nm)	Area %	Area %	Pk 1 (KDa)	Pk 2 (KDa)
To-Et10-30°C	0.22 ± 0.05 ab	468 ± 11 a	649 ± 24 b	94 ± 2 a	96.7 ± 1.9 cd	3.3 ± 0.4 b	1.13 × 10^6^	2.4 × 10^4^
To-Et20-30°C	0.21 ± 0.03 a	474 ± 9 a	575 ± 34 a	100 ± 1 a	98.7 ± 0.6 d	1.3 ± 0.1 a	9.5 × 10^5^	3.7 × 10^4^
To-W-30°C	0.25 ± 0.01 ab	469 ± 12 a	690 ± 38 bc	131 ± 19 b	93.5 ± 2.4 ab	6.5 ± 0.1 c	1.12 × 10^6^	2.5 × 10^4^
To-W-50°C	0.28 ± 0.04 b	450 ± 15 a	654 ± 25 b	121 ± 11 b	95.3 ± 0.2 bc	3.4 ± 0.1 b	8.9 × 10^5^	1.3 × 10^4^
To-W-90°C	0.39 ± 0.02 c	459 ± 2 a	728 ± 15 c	133 ± 1 b	90.8 ± 2.2 a	7.7 ± 0.9 d	1.5 × 10^6^	3.3 × 10^4^
Pe-Et10-30°C	0.26 ± 0.02 a	512 ± 25 c	630 ± 28 b	89 ± 18 a	98.8 ± 0.1 b	1.8 ± 0.5 a	3.3 × 10^6^	3.4 × 10^4^
Pe-Et20-30°C	0.28 ± 0.02 a	437 ± 7 a	607 ± 37 b	84 ± 12 a	98.0 ± 0.1 b	2.0 ± 0.4 a	1.3 × 10^6^	1.4 × 10^4^
Pe-W-30°C	0.25 ± 0.01 a	460 ± 21 ab	499 ± 38 a	nd	98.1 ± 0.4 b	nd	3.8 × 10^5^	nd
Pe-W-50°C	0.25 ± 0.01 a	485 ± 21 bc	544 ± 86 ab	nd	98.2 ± 0.4 b	nd	9.9 × 10^5^	nd
Pe-W-90°C	0.35 ± 0.06 b	490 ± 35 bc	780 ± 39 c	145 ± 15 b	89.8 ± 2.8 a	6.5 ± 1.2 b	2.5 × 10^6^	4.7 × 10^4^

**Table 2:** Percentage content of galacturonic acid (Gal. Ac.), acetic acid, and methanol, and Degree of Methylation (DM) and Degree of Acetylation (DA) expressed as % on dry samples, calculated by ^1^H-qNMR. Values are expressed as mean ± SD from triplicates for each polysaccharide sample. For each series of data, different letters indicate significant differences at *p* < 0.05.

Sample	CH_3_OH	CH_3_COOH	Gal. Ac.	DM	DA
	(%)	(%)	(%)	(%)	(%)
To-Et10-30°C	0.33 ± 0.03 ab	0.59 ± 0.03 b	22.3 ± 0.8 a	8.86	8.57
To-Et20-30°C	0.35 ± 0.03 b	0.81 ± 0.02 c	25.3 ± 0.5 b	8.32	10.42
To-W-30°C	0.15 ± 0.04 ab	0.53 ± 0.01 ab	22.3 ± 0.7 a	4.19	7.72
To-W-50°C	0.11 ± 0.01 a	0.39 ± 0.02 a	33.2 ± 1.1 c	1.98	3.77
To-W-90°C	3.96 ± 0.27 c	2.03 ± 0.20 d	33.8 ± 0.2 c	71.00	19.42
Pe-Et10-30°C	4.64 ± 0.43 b	1.88 ± 0.13 a	44.5 ± 1.2 c	63.05	13.63
Pe-Et20-30°C	3.50 ± 0.02 a	2.46 ± 0.14 b	51.2 ± 0.8 d	53.00	15.53
Pe-W-30°C	4.67 ± 0.33 b	2.70 ± 0.18 b	41.8 ± 1.3 b	67.70	20.87
Pe-W-50°C	4.41 ± 0.04 b	2.53 ± 0.15 b	37.9 ± 0.9 a	70.50	21.57
Pe-W-90°C	3.73 ± 0.03 a	2.05 ± 0.16 a	51.1 ± 0.7 d	61.75	16.25

**Table 3:** List of the extracts obtained by extractions at laboratory scale and by Timatic^®^: L, laboratory scale; T, Timatic; Et10, extraction with 10% of ethanol; Et20, extraction with 20% of ethanol; W, extraction with water; 30, 50, or 90 were the applied temperatures (°C); h, hours; To, tomato; Ba, basil; Pe, red bell pepper.

	Ethanol/Water Extracts		Water Extracts		
	30 °C		30 °C	50 °C	90 °C
Tomato	L-To-Et10-3h	L-To-Et20-3h	L-To-W-3h	L-To-W-2h	L-To-W-2h
	T-To-Et10-3h	T-To-Et20-3h	T-To-W-3h	T-To-W-2h	T-To-W-2h
Basil	-	L-Ba-Et20-3h	-	-	L-Ba-W-2h
	-	T-Ba-Et20-3h	-	-	T-Ba-W-2h
Red bell pepper	L-Pe-Et10-3h	L-Pe-Et20-3h	L-Pe-W-3h	L-Pe-W-2h	L-Pe-W-2h
	-	-	-	T-Pe-W-2h	-

## Data Availability

Data is contained within the article and supplementary material.

## Supplementary Materials

The following supporting information can be downloaded at: https://www.mdpi.com/article/10.3390/ijms242316653/s1.
