# Supplementary material for: Polysaccharides and Phenolic Compounds Recovered from Red Bell Pepper, Tomato and Basil By-Products Using a Green Extraction by Extractor Timatic®
Source: Int J Mol Sci. 2023 Nov 23;24(23):16653. doi: 10.3390/ijms242316653 (PMC10706253; doi:10.3390/ijms242316653)
Supplement: Supplementary file 1 [file ijms-24-16653-s001.zip › ijms-2690070-supplementary.pdf]

SUPPLEMENTARY MATERIAL

.

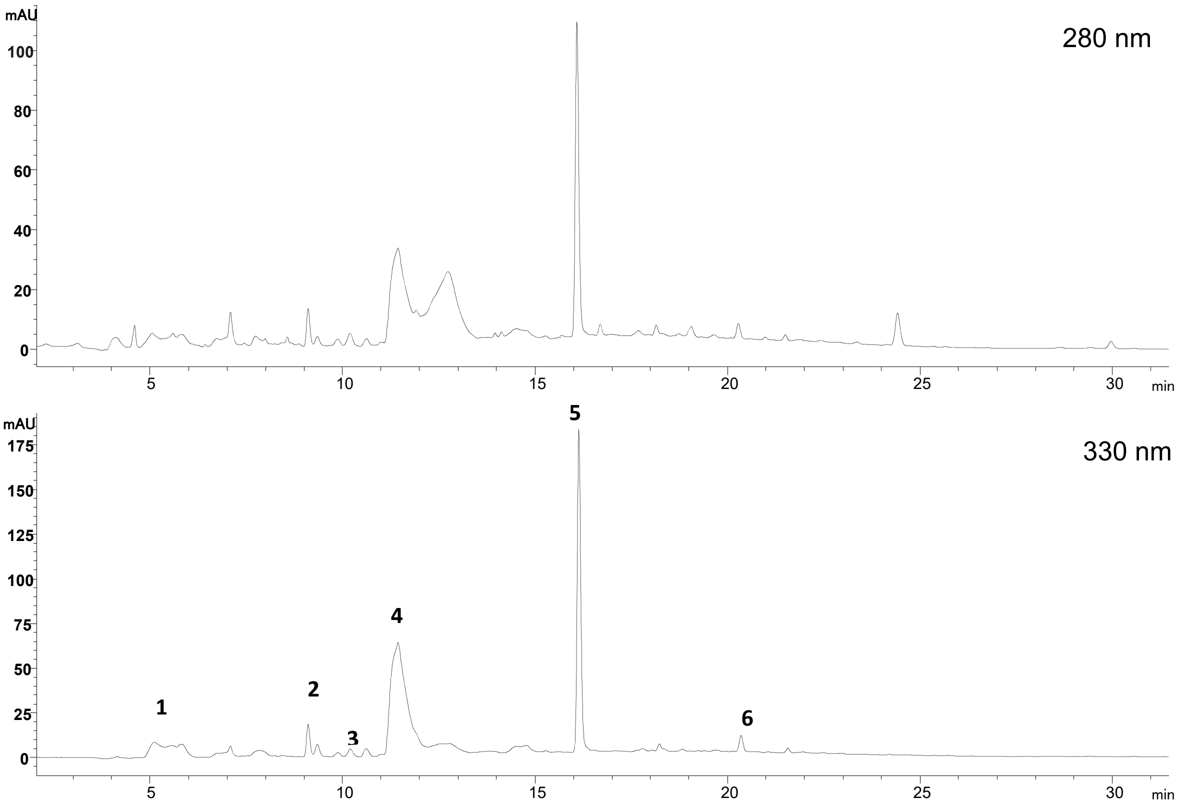

Figure S1. HPLC-DAD profiles at 280 nm and 330 nm of a fresh basil extract. 1, Cinnamic derivatives; 2, Caffeic acid; 3, Cinnamic derivative; 4, Chicoric acid; 5, Rosmarinic acid; 6, Cinnamic derivative

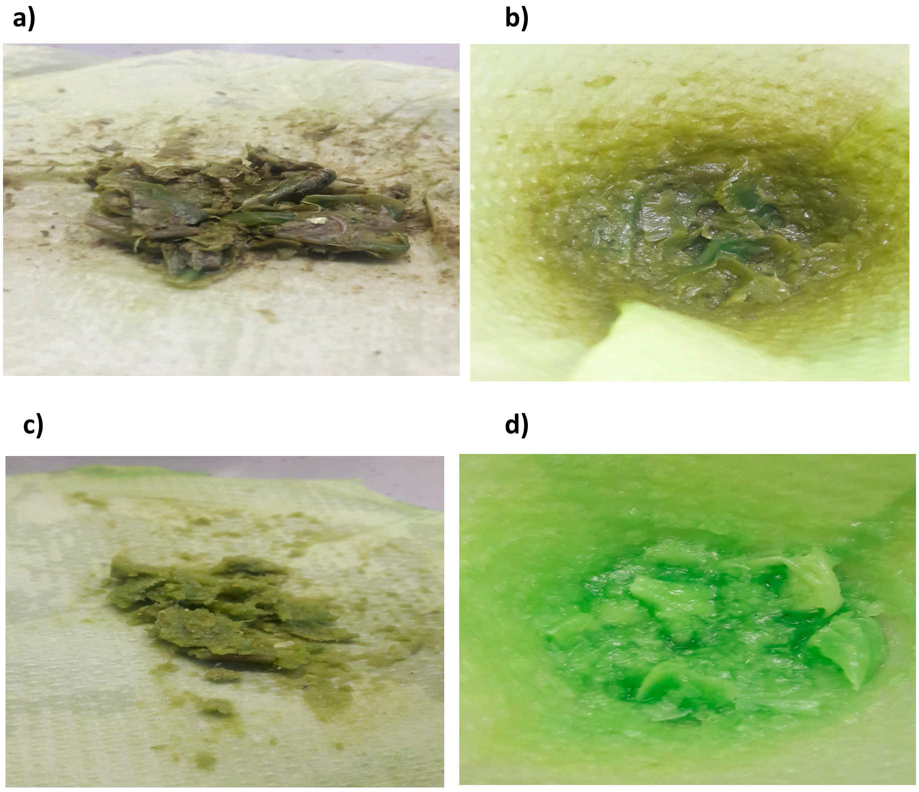

Figure S2. Leaves of basil without pre-treatment with boiling water treated with, **a**) EtOH 70 %; **b**) EtOH 100 %. Leaves of basil pretreated for 30 sec with boiling water and extracted with; **c**) EtOH 70 %; **d**) EtOH 100 %.

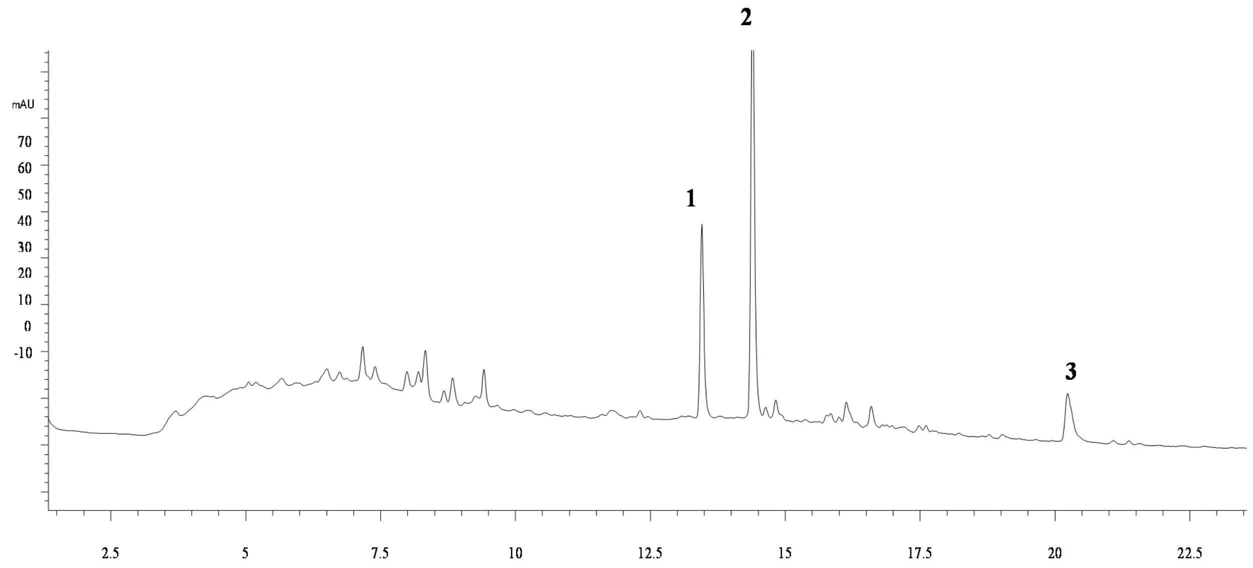

Figure S3. HPLC-DAD profile of tomato extract at 350 nm; the extraction was performed with water at 90 °C. 1, rutin-*O*-pentoside; 2, rutin; 3, naringenin-*O*-hexoside.

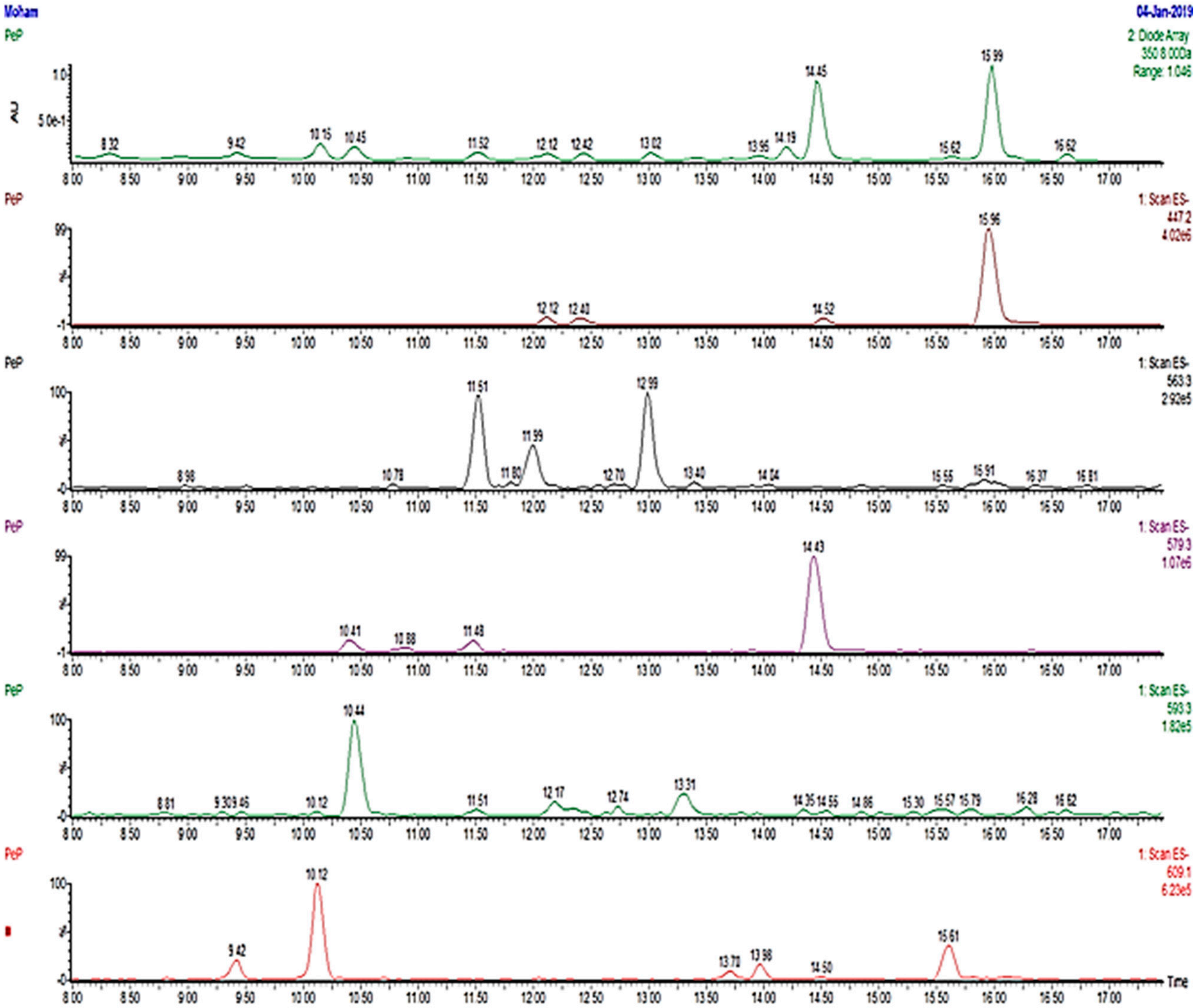

Figure S4. HPLC-DAD-MS (in negative ionization mode) profiles of red bell pepper extract. From top to bottom: profile at 350 nm; EI at 447 m/z (luteolin-*C*-glucoside and isobars); EI at 563 m/z (apigenin-*C*-hexoside-*C*-pentoside and isobars); EI at 579 m/z (luteolin-7-*O*-gluco-pentoside); EI at 593 m/z (chrysoeriol-6-*C*-hexoside-8-*C*-pentoside) and EI at 609 m/z (quercetin-3-*O*-glucoside-7-*O*-glucoside and isobars).
